# Rat tickling: A systematic review of applications, outcomes, and moderators

**DOI:** 10.1371/journal.pone.0175320

**Published:** 2017-04-06

**Authors:** Megan R. LaFollette, Marguerite E. O’Haire, Sylvie Cloutier, Whitney B. Blankenberger, Brianna N. Gaskill

**Affiliations:** 1 Department of Animal Sciences, Center for Animal Welfare Science, College of Agriculture, Purdue University, West Lafayette, Indiana, United States of America; 2 Department of Comparative Pathobiology, Center for the Human-Animal Bond, College of Veterinary Medicine, Purdue University, West Lafayette, Indiana, United States of America; 3 Canadian Council on Animal Care, Assessment and Accreditation Section, Ottawa, ON, Canada; 4 Department of Comparative Medicine, Stanford University, Stanford, California, United States of America; University of Lethbridge, CANADA

## Abstract

**Introduction:**

Rats initially fear humans which can increase stress and impact study results. Additionally, studying positive affective states in rats has proved challenging. Rat tickling is a promising habituation technique that can also be used to model and measure positive affect. However, current studies use a variety of methods to achieve differential results. Our objective was to systematically identify, summarize, and evaluate the research on tickling in rats to provide direction for future investigation. Our specific aims were to summarize current methods used in tickling experiments, outcomes from tickling, and moderating factors.

**Methods:**

We systematically evaluated all articles about tickling identified from PubMed, Scopus, Web of Science, and PsychInfo. Our inclusion criteria were publication in a peer-reviewed journal and collection of original, empirical data on rats using the handling method of tickling. One researcher extracted information from each article. Bias was assessed by 2 investigators using the SYRCLE bias assessment tool.

**Results:**

We identified 32 articles (56 experiments) published in peer-reviewed journals about rat tickling for inclusion. A wide variety of strains, sexes, and ages of rats were included. The most common method used for tickling was cycling through 15 seconds of tickling and 15 seconds of rest for 2 minutes for 3 to 5 days. Experiments with a control for tickling (N = 22) showed that tickling increases positive vocalization, approach behavior, decreases anxiety measures, improves handling, and in some cases decreases stress hormones. Tickling juvenile, individually housed rats with a trait predisposition to respond more positively to tickling, results in the most positive outcomes. Methods to reduce bias were insufficiently reported.

**Conclusions:**

We conclude that tickling is a promising method for improving rat welfare and investigating positive affect. However, the establishment of tickling best practices is essential as the outcomes from tickling can be moderated by several factors.

## Introduction

### Importance

The study of positive affect has previously been difficult to model and study in laboratory animals. Understanding positive affect has wide-reaching importance for humans and animal welfare. Positive affective states in humans confer resilience to depression and anxiety as well as lead to an overall increase in general health [1]. Therefore, modeling positive affect in animals could further human health research. Measuring and eliciting positive affect/experiences are also important for assessing animal welfare [2]. The technique of tickling, and measuring positive vocalizations in rats, is a promising solution to modeling and quantifying positive affective states in laboratory animals [3]. Additionally, by increasing positive affect and decreasing rat fear of humans, tickling may be a promising technique to improve rat welfare [4].

### Current knowledge

The technique of tickling rats was developed by Dr. Jaak Panksepp and Dr. Jeffrey Burgdorf in 2000. The aim of their initial publication was to determine if a “tickling-type somatosensory stimulation by a human hand” that mimicked juvenile rat rough-and-tumble play was rewarding to rats. In their original article involving eight experiments, juvenile Long Evans rats were tickled for two minutes across four to ten total sessions. The tickling technique involves two main components: a “dorsal contact” on the rat’s neck and a “pin” on the rat’s stomach. Developing this technique was additionally used to investigate the possibility that 50-kHz ultrasonic vocalizations, produced by rats during play and other rewarding situations, could be used to measure reward value and emotion in rats. Subsequently, vocalizations in the 50-kHz range have repeatedly been shown to be good indicators of positive affect and welfare of rats [5, 6]. Some indications that 50-kHz vocalizations are positive are that they are produced in a range of contexts such as during play, mating, exploratory activity, amphetamine administration, and in anticipation of food rewards [7]. These vocalizations can be recorded by using specialized microphones and later quantified by trained observers. In contrast to 50-kHz calls, vocalizations in the 22-kHz range have been demonstrated to reflect negative affect and are produced during exposure to predators, pain, and inter-male fighting [5].

### Rationale

Since the initial publication of the tickling technique, a number of laboratories have used tickling to investigate and measure positive affect [8] as well as decrease rat stress, and habituate rats to human contact [4]. However, tickling experiments use a range of protocols that find a wide variety of outcomes. Neither tickling protocols nor specific outcomes from tickling that may improve rat welfare have been summarized. Additionally, a number of experiments have also specifically investigated factors that may moderate the outcomes of tickling. However, without a singular article describing these factors, it is unclear how tickling is being used and what factors pertaining to rat characteristics (strain, sex, age) and the tickling protocol (session duration, total number of sessions) influence the efficacy of tickling. As a result, researchers are left with insufficient guidance for using tickling either as a tool for modeling and measuring affect or to improve rat welfare in their experimental colonies. This lack of guidance may also prevent the widespread adoption of this technique for general enrichment since researchers and managers of animal care programs are unsure what time investment this technique requires or its potential effects on their research.

### Objectives

Previously, meta-analyses and systematic reviews of animal studies have proven useful for optimizing study design and reporting [9]. Although there have been reviews of using tickling and ultrasonic vocalizations to model laughter in humans [10, 11], there has not yet been a systematic review of how tickling is utilized in research, its outcomes related to affect and welfare, and methodological considerations for its use. Therefore, the purpose of this review was to provide a comprehensive overview of the empirical research using the tickling technique in rats. Our objective was to systematically identify, summarize, and evaluate the research on tickling in rats to provide direction for future investigation. Our specific aims were to (a) describe current methods used in tickling articles, (b) describe outcomes from tickling, and (c) summarize factors that have the potential to alter tickling outcomes. By summarizing this information in one location, it is possible for researchers, laboratory personnel, those interacting with pet rats, and other interested individuals to make educated decisions about the use and application of tickling.

## Methods

### Protocol

Before beginning our review, we consulted the preferred reporting guidelines for systematic reviews and meta-analysis (PRISMA) guidelines [12, 13], the Systematic Review Centre for Laboratory Animal Experimentation (SYRCLE) E-learning course and guides, and a veterinary science librarian. We defined our procedures a priori in an unpublished study protocol that specified the search strategy, inclusion and exclusion criteria, and data extraction items.

### Article identification

We identified articles by searching complete electronic databases from their first index date through August 2016 and scanning reference lists of identified articles. Databases included Scopus (1966—Present), PubMed (1946—Present), Web of Science (1900—Present), and PsycINFO (1840-Present). Titles, abstracts, and keywords were used for the search. Search terms for all databases included at least one identifier for tickling and rat or rats. Identifiers for tickling included playful handling OR tickle OR tickling OR heterospecific play. No language restrictions were applied. See **S1 Text** for the full search strategy for each database.

### Article selection

After removing duplicate articles, we screened all references for inclusion based on their title and abstract. If we could not determine inclusion by title and abstract, we reviewed the full-text manuscript. We used the following inclusion criteria, in order, to select relevant articles for review: (1) publication in a peer-reviewed journal, (2) collection of original, empirical data, (3) on rats (4) using the handling method of tickling. Collection of original, empirical data is defined as the systematic collection and reporting of original observational or experimental scientific data. All articles were independently assessed by two reviewers (MRL and LMF). Disagreements were resolved via discussion.

### Data extraction

One researcher (MRL) extracted information from each included article to achieve the three aims of this review. If the article reported data from several experiments, information was extracted individually for each experiment. In this review, “article” refers to the entire paper while “experiment” refers to a singular experiment within an article. To achieve the first aim–describe the current use of tickling–we extracted data items from all experiments including terminology, aim, design, rat age, sex, strain, and housing; and tickling duration, frequency, total time, and location factors. To achieve the second aim–describe outcomes from tickling–experiments were identified that used a control group specific to the tickling intervention, such as stroking. To achieve the third aim–summarize the factors that have the potential to alter tickling outcomes–experiments were identified that described using variants of a tickling technique or the same tickling technique in different situations or animals. Data items for the second and third aims included type of control or comparison condition, outcomes measured, and whether each outcome was increased or decreased. An outcome was coded as increased or decreased based on the conclusion of the author in the original manuscript. Outcomes were organized by type from most- to least-commonly reported. Effect sizes and confidence intervals were not commonly reported within studies and therefore were not extracted as data items. We also extracted additional data about the publications including first author, publication year, journal name, country, and title.

### Reporting quality and risk of bias assessment

Two independent reviewers (MRL and WBB) assessed reporting quality and risk of bias of each experiment included within each article. Reporting quality was assessed by answering one question: did the experiment report any sort of randomization of rats to treatment groups in the methods section. Risk of bias was assessed using a modified version of the Systematic Review Center for Laboratory animal Experimentation (SYRCLE) Risk of Bias Tool for animal intervention studies [14]. The SYRCLE Risk of Bias Tool is adapted from the Cochrane Collaboration Risk of Bias Tool [15] to address bias specific to animal experiments. In brief, the tool asks reviewers to assess 5 domains of bias including selection bias, performance bias, detection bias, attrition bias, and reporting bias. When assessing selection bias, we considered groups similar at baseline if their number of ultrasonic vocalizations were not statistically different. When discrepancies between reviewers occurred, we reached consensus by discussion.

Three main modifications were made to this tool. First, we split item 5 Blinding Caregivers/Investigators into two parts to assess the blinding of caregivers and investigators separately. This was done because although no studies indicated blinding caregivers, a few studies did blind investigators. Second, we added two potential answers to the bias questions: not applicable or partial. These answers were added to accommodate the use of this tool on a wide variety of experimental designs beyond the traditional randomized control trials. The answer “not applicable” was used when studies applied the same treatment to all animals in the study, therefore making certain techniques to address bias not relevant. The answer “partial” was used for randomization reporting and item 7a to indicate a medium risk of bias when only part of the treatment group was reported as randomized or if not all outcomes were blinded during assessment. Third, we added question 7b to indicate which outcomes were blinded (if any) as a follow-up question on medium risk of bias answer.

## Results

### Article identification and selection

**Fig 1:**
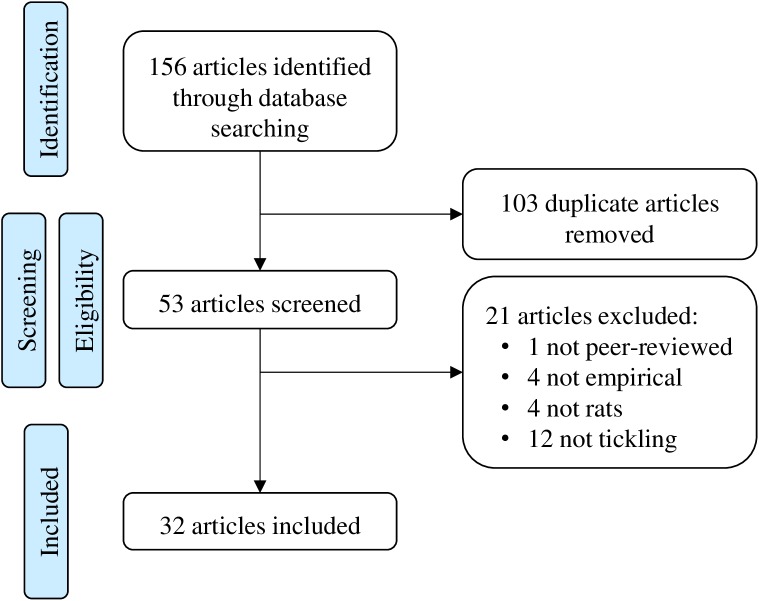
Article Selection. A flow chart of the selection process of articles using tickling in rats from an original search of databases.

The literature search resulted in 156 citations. A flowchart of the article selection process is presented in **Fig 1**. The final sample included 32 articles (20.5% of the total initial pool) published between 2000 and 2016 that met the inclusion criteria of empirically evaluating outcomes from tickling rats. There was an international representation of researchers including corresponding authors from the North America (n = 14), Europe (n = 12), and Asia (n = 6). The articles were published in a variety of disciplines, primarily related to Neuroscience (n = 14), Behavior, Physiology, or Psychology (n = 13), or Pharmacology (n = 5). We focused this review on descriptive and qualitative synthesis, rather than meta-analysis, because of the large variety of study designs, animals, intervention techniques, reported outcomes, and possibility of bias.

### Characteristics of tickling

To achieve our first aim–to describe the characteristics of tickling–we extracted and summarized the key features and applications across the 56 experiments of the 32 articles. All results are presented by experiment in **S1 Table**.

#### Terminology

All 32 articles used the word “tickling” at least once although some specified it as “manual tickling,” “tickling stimulation,” or “tickling-like stimulation”. Preferred and additional terms varied across articles. These additional terms most commonly included words such as play or playful (n = 11), heterospecific (n = 9 terms), somatosensory (n = 3), tactile (n = 5), stimulation (n = 4), or wrestling (n = 1). The most common additional term used was heterospecific play (n = 6) followed “playful handling” (n = 4). Nine articles only used one term while the remaining 23 used multiple terms.

#### Tickling methodology

A variety of methods for tickling were used within each article (Table 1) of which a summary of tickling dosage is presented in Table 2. The most common (46%, n = 26) dosage and technique of tickling used was to replicate the “Panksepp Method” of 15 sec baseline rest followed by 15 sec of dorsal contacts and pins for a total of 2 minutes for at least 4 days [16]. The second most common (39%, n = 22) tickling dosage was to use a “Panksepp Variation” which involves the same distinctive dorsal contact and pin components with 15 sec intervals, but with a different session length or number of days. A few experiments (9%, n = 5) also used the “Schwarting Method” which involves using a variety of specific techniques (neck tickle, hand chase, grab and tickle, etc.) with random breaks for at least 3.5 minutes for at least 3 days [17].

**Table 1:** Specific Methods Used for Tickling Rats.

First Author	Year	Type	Total Duration(min)	Active Tickling (min)	# of Sessions	Total time per rat (min)	Tickling Bedding
Boulay	2013	PV	?	0.5	1–6	?	Sawdust
Brudzynski	2011	P	2	1	4	8	Corn cob
Burgdorf	2001	P	0.5–2	0.15–1	2–5	8–10	None
	2005	P	2	1	4	8	Corn cob
	2008	PV	2	1	1	2	None
	2009	P	2	1	5	10	None
Cloutier	2008	PV	1	0.5	10	10	None
	2012	P	2	1	15, 17	30, 34	Wood fiber
	2013	P	2	1	21	42	Wood fiber
	2014	P	2	1	30	60	Wood fiber
	2015	P	2	1	10	20	Wood fiber
Garcia	2015	P	2	1	4	8	Wood fiber
	2016	P	2	1	4	8	Wood fiber
Hori	2009	PV	5	2	20	100	Cloth
	2013a	PV	5	2	1	5	Cloth
	2013b	PV	5	2	10	50	Cloth
	2014	PV	5	2	20	100	?
Koiv	2016	P	2	1	14	28	None
Mallo	2007	P	2	1	23, 46	46, 92	?
	2009	P	2	1	14	14	None
Natusch	2010	S	10	7	2, 5	20, 50	Aspen, None
Panksepp	2000	P	2, 5	1, 2.5	4–8	14–35	Corn cob
Paredes-Ramos	2012	PV	6	3	10	60	None
Popik	2012	PV	0.5	0.5	24	?	?
	2014	PV	?	0.5	14	?	?
Raudkivi	2012	P	2	1	14	28	None
Roccaro	2016	S	3.5	5	12	60	None
Rygula	2012	P	0.5	0.5	1	1	?
Schwarting	2007	S	10	7	3	30	Aspen
Wöhr	2009	S	10	4.5	5	50	Aspen
Yamamuro	2010	P	5	2	5	10	Cloth
	2013	P	5	2	14, 28	28, 56	Cloth

Key characteristics about the specific methods used in the 32 articles included in the analysis. Type indicated the type of tickling. (P = Panksepp, PV = Panksepp Variation, S = Schwarting), total duration of each tickling session, the time within that session that the researcher was actively tickling the rat (most sessions include rest periods during which the rat is not tickled), the total number of sessions, the total time investment per rat, and the type of ground cover/bedding. When articles had 2 experiments with different values those values are split by a comma. Articles with >2 experiments with different values have a dash indicating the range of values used.? = not reported.

**Table 2:** Tickling Dosage.

Variable	Mean	SD	Min	Max	Mode
Session Length (min)	3.3	2.5	0.5	10	2
Active Tickling (min)	1.7	1.6	0.15	7	1
Number of sessions	9.4	8.5	1	46	5
Total time investment per rat (min)	27.4	25.7	1	100	10

A summary of the specific procedures used for tickling rats across 56 experiments within 32 articles using tickling.

Tickling occurred in a variety of locations, times of day, light phase, and lighting condition. A majority of experiments reported the flooring of the cage the rats were tickled in (87%, n = 46), including bedding (n = 21), uncovered cage bottom (n = 18), cloth (n = 5), or both bedding and empty flooring (n = 2). Rats were usually tickled individually in a test cage or box (77%, n = 43) although a few experiments tickled rats in their home cage (11%, n = 6) or did not report where rats were tickled (7%, n = 4). Time of day of tickling was also reported for less than a half of experiments (41%, n = 17); this included during the light phase (n = 18) or during the dark phase (n = 3). Less than one third of experiments reported lighting conditions during tickling (21%, n = 12); these included red lighting (n = 6), a light intensity of 150 lux (n = 4), dim (n = 1) or “normal” (n = 1).

#### Article characteristics

Key article characteristics are summarized in Table 3. Tickling was applied to the four of the commonly used rat strains including Long-Evans (39%, n = 22), Sprague-Dawley (29%, n = 16), Wistar (23%, n = 13), and Fisher (5%, n = 3; 2 experiments did not report strain). More than half of experiments investigated tickling only in male rats (57%, n = 32), followed by using both sexes (32%, n = 18) and very few used only females (7%, n = 4; 2 experiments did not report which sex was used). Most experiments tickled juvenile rats less than 6 weeks (68%, n = 38), although some experiments tickled adult rats older than 6 weeks (25%, n = 14; 4 experiments did not report rat age).

**Table 3:** Rat and Housing Characteristics of Tickled Rats.

First Author	Year	N	Strain	Sex	Age (days)	Rats per cage	Acclimation (days)
Boulay	2013	6–16	SD	M	21	4	1
Brudzynski	2011	66	LE	M	21	1	?
Burgdorf	2001	8–49	LE	M & F	37	1	?
	2005	240	LE	M & F	24	1	3
	2008	9	LE	M & F	31.5, 180	1, 3–5	?
	2009	18–83	LE	M & F	24–126	1	N/A
Cloutier	2008	40	SD	M	65	2	14
	2012	16, 32	SD	M	35, 57	1	?
	2013	72	SD	M	21	1, 2, 3	3
	2014	48	SD	M	25	1, 2, 3	4
	2015	96	SD	M	32	1	11
Garcia	2015	20, 30	SD	M	40	1	10
	2016	30	SD	M	40	1	10
Hori	2009	8	Wistar	M	28	1	7
	2013a	12	Fisher	M	37.5	1	5
	2013b	79	Fisher	M	25	1	14
	2014	95	Fisher	M	21	1, 3	5
Koiv	2016	40	Wistar	M	22	4	?
Mallo	2007	29, 58	Wistar	M & F	22	1, 4	0
	2009	62	Wistar	M & F	21	1, 4	0
Natusch	2010	12	Wistar	M	70	6	3
Panksepp	2000	12–48	LE	M & F	28–50	1, 2	?
Paredes-Ramos	2012	20, 30	?	F	31, 92	1	?, 5
Popik	2012	40	SD	M	?	4	7
	2014	33	SD	M	?	5	7
Raudkivi	2012	40	Wistar	M	21	1, 3–4	0
Roccaro	2016	37	Wistar	M & F	21	1	?
Rygula	2012	26	SD	M	?	4	7
Schwarting	2007	20	Wistar	M	?	1	4
Wöhr	2009	18	Wistar	M	49	1	3
Yamamuro	2010	12	Wistar	?	28	1	7
	2013	70	Wistar	?	28	1	7

Key characteristics about the rats used and their housing from 56 experiments from 32 articles about rat tickling. Rats per cage indicates the number of rats that were housed in each cage during the experiment. When articles had 2 experiments with different values those values are split by a comma. Articles with >2 experiments with different values have a dash indicating the range of values used.? = unclear or not reported. N/A = not applicable

More than half of the experiments (55%, n = 31) housed their rats individually though some (20%, n = 11) housed their rats in groups for the entire experiment and others (25%, n = 14) either housed at least some rats socially for part of the experiment. Most experiments (71%, n = 40) housed their rats in “standard” plastic cages. However, the original tickling experiments (23%, n = 13) housed rats in wire mesh cages with wire mesh flooring [16, 18]. Three experiments did not report the type of caging their rats were housed on.

Total sample sizes ranged from 8 to 240 with an average of 35 rats. Rats in experiments that reported acclimation days (59%, n = 33) received anywhere from 0 to 29 days with an average of 5.5 days. In general, articles did not report what activities were done during acclimation, however one article noted that all rats were “handled” for 5 minutes per day for 3 days during acclimation [19].

### Outcomes of tickling

**Fig 2:**
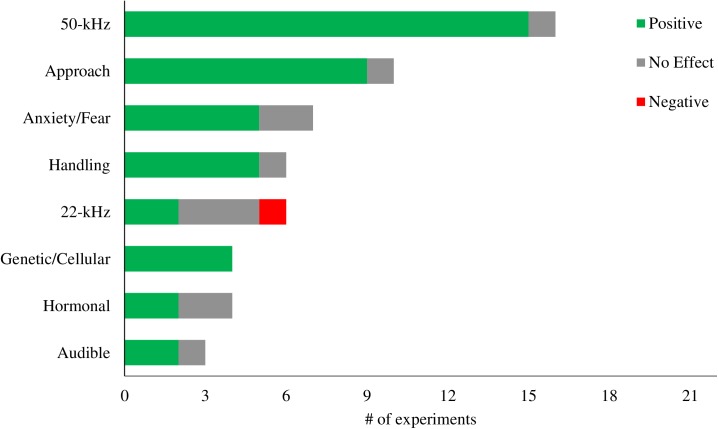
Outcomes from Tickling. The impact of tickling on various outcomes assessed by experiments with a control specific to tickling (N = 22). Green = positive results from tickling. Grey = no difference from tickling. Red = negative result from tickling.

To achieve our second aim–to describe the outcomes of tickling–we extracted and synthesized outcomes from all experiments that included a control comparison to tickling (22 experiments from 17 articles). Although the specific designs and assessments of these experiments varied, we identified key types of outcomes and categorized them according to the number of experiments in which they were reported (**Fig 2; Table 4**). Types of control/comparison treatments in each experiment included minimal handling (n = 11), light touch or stroking (n = 7), transfer to test box without tickling (n = 3), restrained on back (n = 3), exposure to a passive hand (n = 2), or food treat (n = 1). Seventeen experiments only used one control/comparison group while four experiments used multiple control/comparison groups.

**Table 4:** Tickling Outcomes

			↑ = Positive		↓ = Positive		
First Author	Year	Control Group or Condition(s)	50s	Human Approach	Anxiety & Fear	Handling Reactivity	22s
**Burgdorf**	2001	Light touch; Minimal	↑	↑			
**Cloutier**	2008	Food treat; Stroking	-	-	-	-	
	2012	Minimal; Passive hand; Restraint	↑	↑	-	↓	-
	2013	Minimal	↑		↓	↓	-
	2014	Passive hand	↑			↓	↑
	2015	Minimal	↑			↓	-
**Hori**	2009	Light-touch	↑	↑			↓
	2013a	Light-touch	↑	↑			↓
	2013b	Minimal	↑	↑	↓		
	2014	Minimal	↑	↑	↓		
**Mallo**	2007	Minimal			↓		
**Panksepp**	2000	Minimal	↑				
**Paredes-Ramos**	2012	Minimal					
**Rygula**	2012	Minimal	↑				
**Wöhr**	2009	Minimal					
**Yamamuro**	2010	Minimal	↑	↑			
	2013	Minimal; Light-touch	↑	↑			

A list of the most commonly assessed outcomes (in at least 5 experiments) of tickling rats compared to at least one control group or condition. Arrows indicate the direction of the results as either higher or lower than control rats. A dash indicates that this measure was assessed but no effect or a mixed effect was found. 50s = 50 kilohertz ultrasonic vocalizations (indicative of positive affect). 22s = 22 kilohertz ultrasonic vocalizations (indicative of negative affect). Minimal = minimal handling. Passive = exposure to a passive, still hand. On the left side of the bold line it is considered positive for these outcomes to increase and on the right side of the line it is considered positive for these factors to decrease

#### Vocalizations

The most commonly assessed outcome from tickling was production of 50-kHz vocalizations, evaluated in 72% (n = 16) of tickling controlled experiments. Nearly all experiments (94%, n = 15) found that tickled rats produced more 50-kHz vocalizations than control rats. The one experiment that found no effect tickled rats directly after injection [20].

Six experiments assessed 22-kHz vocalizations and found mixed results: 33% (n = 2) found fewer 22-kHz vocalizations, 50% (n = 3) found no effect, and 17% (n = 1) found more 22-kHz vocalizations. The two experiments that found an effect of handling treatment used a light touch comparison condition [21, 22]. The three experiments that found no effect of tickling treatment used a minimal handling, restraint, or passive hand comparison condition [4, 23, 24]. The experiment that found that tickled rats produced more 22-kHz vocalizations specifically found that only the rats that had been tickled as adults, not juveniles, produced more 22-kHz calls and that all rats were tickled after an injection [25].

Three experiments assessed audible vocalizations and found mixed results. One experiment found no effect on audible vocalizations during injection [20]. Two experiments found tickled rats made fewer audible vocalizations during an injection [24, 25]. However, one of these experiments also found that during the actual tickling procedure, tickled rats made more audible vocalizations [24], while the other experiment found no effect of tickling treatment on audible vocalizations during the tickling procedure [25].

#### Approach behavior

Ten experiments (48%) assessed outcomes of tickling on approach behavior. Among these, 90% (n = 9) found positive results such as decreased approach latency. Two different methods for evaluating approach were used among the ten experiments: 70% (n = 7) evaluated rats directly after tickling and 30% (n = 3) conducted a human approach test delayed from tickling by three days. Testing rats directly after tickling always led to positive results. Testing rats 3 days post tickling has mixed results: 1 found no effect of tickling [20] and the other 2 found tickled rats reared, nibbled, and made more contacts with the hand than controls [4].

#### Other anxiety and fear behaviors

Seven experiments (33%) assessed the outcomes of tickling on anxiety and fear behaviors during standardized tests. Among these, 71% (n = 5) found fewer anxiety and fear behaviors in tickled rats. Three distinct positive results were found: decreased freezing after fear conditioning [22, 26], increased locomotor activity and entries into the center of an open field arena [23], and increased entries and time in the open arms of an elevated plus maze by high-callers only on day 2 of testing [27]. However, elevated plus maze behavior was unaffected by tickling in two other studies [4, 20]. Additionally, no behavioral changes due to tickling treatments were observed in a Forced Swim Test [27], Cat Odor Test [4], Flinch-Jump Test [26], or Morris water maze [26].

#### Handling effects

Six experiments (29%) assessed outcomes of tickling on the stress associated with handling. Among these, 83% (n = 5), found reduced stress in tickled rats. Five experiments investigated the effects of tickling on the injection experience. Four of these found that tickling increased 50-kHz vocalizations while decreasing approach latency, audible calls during injection, and injection procedure duration [4, 24, 25]. The experiment that found no effect, tickled rats *after* injection only [20]. Later research showed that tickling rats only before or before *and* after injection, particularly combined with juvenile tickling experience, is more rewarding than tickling rats after injection [24]. Additionally, tickling increases 50-kHz calls in anticipation of handling [23].

#### Hormonal effects

Four experiments (19%) assessed effect of tickling on rat hormones. Among these, 50% (n = 2) found at least some positive effects and 50% (n = 2) found no effects. Tickling increased dopamine release in the nucleus accumbuens [28] and decreased adrenaline and noadrenaline after fear conditioning [22]. However, tickling was never found to effect corticosterone in the feces [23, 25] or blood [22].

#### Genetic and cellular level effects

Four experiments used tickling of rats to elicit a positive emotional state for studying the effects of positive affect on cell proliferation, neurogenesis, or gene expression. Tickling increased adult hippocampus cell proliferation, but only in high-callers [19]. Adult hippocampus cell proliferation is a factor associated with depression. Tickling altered the expression of genes in the hypothalamus related to feeding regulation [21]. This experiment was an attempt to model and investigate the effect seen in humans that blood glucose levels improve after laughter. [21]. Tickling increased neurogenesis in the dentate gyrus of the hippocampus [29]. The dentate gyrus is an area thought to mediate learning and memory formation. Tickling up-regulated production of kallikrein family mRNAs [30], which is one of the serine proteases and related to the production of many bioactive peptides in the submandibular gland of the rat. These authors believe it could be a candidate for a biochemical marker of positive emotional state [30].

#### Other outcomes

Two experiments reported outcomes that have not been replicated. Tickling high-calling rats from normal lines induced a positive cognitive bias compared to no tickled or tickled low-calling rats [8]. Tickling adult rats was unsuccessful in inducing a conditioned place preference or sexual partner preference based on the smell of the hand that tickled them [31].

### Tickling outcome moderation

To achieve our third aim–to summarize factors that could moderate tickling outcomes–we extracted and summarized the key outcomes from experiments that investigated factors that could moderate the results of tickling (40 experiments from 24 articles).

#### Inter-individual differences in randomly bred rats

Inter-individual differences in randomly bred rats as a moderator of tickling was evaluated in 30% (n = 12) of moderator experiments. All experiments reported at least some significant effects on outcomes which are shown in **Table 5**

**Table 5:** Inter-Individual Differences in Tickling Outcomes.

Results of high-calling trait	N	Author Year
↓ susceptibility to chronic variable stress	5	Wohr 2009; Mallo 2007; Mallo 2009; Raudkivi 2012; Koiv 2016
↑ 50-kHz vocalizations in open field test	1	Garcia 2015
↑ 50-kHz vocalizations during amphetamine treatment	1	Garcio 2016
↓ anxiety/fear behaviors (more crosses in central area of open field and more rearing/locomotor activity to tickling)	2	Wohr 2009; Burgdorf 2009
↑ hippocampal cell proliferation after tickling	1	Wohr 2009
↓ approach time to self-administer tickling	1	Burgdorf 2008
↑ positive cognitive bias after tickling	1	Rygula 2012

Randomly bred rats that consistently produce more 50-kHz vocalizations during tickling (high-callers) have been found to have the following results when compared to rats consistently vocalizing less during tickling (low-callers). N = number of experiments with this finding.

Varied methods were used to group rats according to inter-individual differences, although all experiments used 50-kHz vocalizations. Five experiments (42%) used a median split of their average response on the 12-14^th^ day of tickling [27, 32–34]. Four experiments (33%) used calling rate as a continuous covariate [19, 35, 36]. One experiment used total 50-kHz vocalizations after just 1 session of tickling [8]. One experiment split rats into upper and lower quartiles [37]. One experiment did not detail the methods of separation [38]. Also, one experiment investigated vocalization subtypes and found that only frequency modulated 50-kHz vocalizations differed between groups, but that flat 50-kHz vocalizations and 22-kHz vocalizations were not different [37].

#### Pharmacology

The impact of pharmacological substances on the production of 50-kHz vocalizations during tickling was evaluated in 20% (n = 8) of experiments investigating tickling moderation. Results on 50-kHz are presented in **Table 6.**

**Table 6:** Pharmacological Impacts on 50-kHz Vocalizations.

Effect	Drugs	Author year
Increase	Buspirone or SSR181507 after Phencyclidine application	Boulay 2013
	Metyrapone + Stress (compared to Vehicle + Stress)	Popik 2014
	*Naloxone* for socially housed rats	Burgdorf 2001
	*Amphetamine*	Koiv 2016;
Decrease	Phencyclidine	Boulay 2013
	*Naloxone* for individually housed rats	Burgdorf 2001
	MK-801	Panksepp 2000
	SCH23390 or Racolpride	Hori 2013
No effect	Quipazine, Cyprohetadine, Morphine, Scopolamine, *Amphetamine*, or Haloperidol	Panksepp 2000
	Aripiprazole, Eplivanzerin, SSR103800, SSR181507, Diazepam, Buspirone, or Fluoxetine	Boulay 2013
	Aripiprazole, Eplivanzerin, SSR103800, Diazepam, or Fluoxetine after Phencyclidine application	Boulay 2013

A summary of the impact of various drugs on the production of positive 50-kHz vocalizations produced by rats during tickling. Italics indicate drugs in which mixed results have been found.

#### Housing

The influence of housing type (solitary versus group) on tickling outcomes was evaluated in 20% (n = 8) of experiments investigating tickling moderation. Among these, 88% (n = 7) reported more positive outcomes in solitary rats compared to group-housed rats. Specifically, solitary rats produced more 50-kHz vocalizations during tickling [16, 18, 37], had shorter approach latencies [16, 18], and had more play bites [16]. Conversely, socially housed rats learned to avoid tickling during an instrumental learning task [16]. However, 22-kHz vocalizations were not found to differ between housing types [37]. No significant differences were found between solitary, pair, or triplet housed rats in 50-kHz or 22-kHz vocalizations in one experiment [23]. A key methodological difference with this experiment was that group housed rats were tickled with their cage mates in their home cage, rather than tickled singly in a test cage [23].

#### Genetics

The ability to select rats for their response to tickling, and the subsequent effects of this selection, was evaluated in 18% (n = 7) of experiments investigating tickling moderation. Three experiments successfully bi-directionally selected rats to exhibit high or low levels of 50-kHz vocalizations in response to tickling based on their call rate on the 4^th^ day of tickling [16, 38, 39]. All experiments found that high-line rats produced more 50-kHz vocalizations as compared to random-line or low-line rats [16, 38–40]. Overall high-line rats seem to have a stress-resilient phenotype while low-line rats seem to have a stress prone phenotype. Specific results are presented in **Table 7**.

**Table 7:** Differences in Tickling Outcomes in Bi-Directionally Selected Rats in Comparison to Randomly Bred Rats.

Outcome Measurement	High-Line*	Low- Line*	Author Year
Approach behaviors after tickling	↑	-	Panksepp 2000
Tickling avoidance	↓	-	
Fecal boli during tickling, open field test, & porsolt swim test	-	↑	Burgdorf 2009
Bites towards resident animal in social defeat test	↓	-	
Contact with conspecifics in a social interaction test	-	↓	
Preference for dilute sucrose	↑	-	
Anxiety & Fear Behaviors during Open Field	↓	↑	
Level of metenkephalin in brain	↑	↓	
Level of cholecystokinin in brain	↓	↑	
Spontaneous locomotor activity	↑	-	Brudzynski 2011

The effects of bi-directional selection of production of high or low levels of 50-kHz 50-kHz vocalizations during tickling (high-line or low-line). *All results are in comparison to randomly bred rats. A dash indicates no significant difference compared to randomly bred rats.

#### Other moderations

Ten experiments investigated additional factors that can moderate tickling outcomes including bedding, age, brain lesioning, and tickling timing and technique. Using bedding in the tickling cage increased the reward value of tickling and number of positive vocalizations as compared to no bedding [41]. The impact of age is nuanced. Younger juvenile animals produce more positive vocalizations and show higher reward value in one study [31], but another experiment shows no difference in 50-kHz or 22-kHz vocalizations [38]. However, adult rats tickled as both juveniles and adults show the most positive results as compared to rats only tickled a juveniles or adults [25]. Rats tickled as both juveniles and adults produced more 50-kHz vocalizations before and after an injection as an adult, fewer audible calls during injection, and the injection procedure was shorter as compared to tickling rats only as juveniles or adults. Tickling rats 1 hour after restraint stress reduces 50-kHz vocalizations [42, 43], while tickling rats 23 hours after restraint stress has no effect on 50-kHz vocalizations [42]. Tickling rats after lesioning the parvafox nucleus also decreases 50-kHz vocalizations and approach behavior [44].

Tickling outcomes are also moderated by tickling timing [24] and technique [17]. Tickling before injection is more effective than tickling after injection [24]. Different techniques investigated included chasing the rat with a hand at its rear, holding the rat in one hand while tickling its stomach with the other, tickling the belly without flipping the rat over, pressing with one finger between the shoulder blades, and allowing the rat to chase the experimenter’s hand. This tickling method had a session duration of 10 minutes per day for 3 days. Per visual inspection of the results, techniques of neck tickle, hand chase, grab & tickle, and push & drill (pressing with one finger between the shoulder blades) evoked the most 50-kHz calls while neck tickle and full back (tickling the entire back) evoked the most 22-kHz calls. However, the “Schwarting Method” of tickling evokes significantly higher 22-kHz calls (227 ± 38.4 (0–420) 22-kHz vocalizations) in comparison to the “Panksepp Method” which typically evokes 10 or fewer 22-kHz vocalizations per minute of tickling

#### Reporting quality and risk of bias assessment

**Fig 3:**
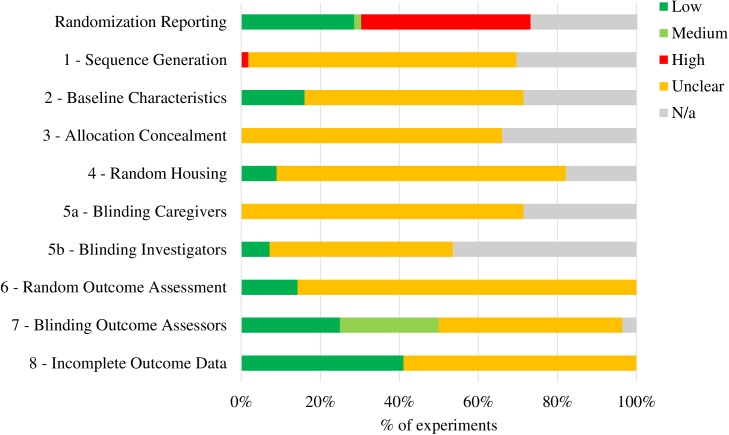
Reporting Quality and Risk of Bias. Reporting Quality was assessed by answered the question if any sort of randomization was reported in the methods section. Risk of Bias was assessed using a modified version of SYRCLE Risk of Bias Tool in 55 experiments that used rat tickling. Unclear risk indicates that studies did not report whether bias was mitigated or not. Low risk indicates there was proper bias mitigation. Medium risk indicates that only part of the randomization was reported or outcome assessment was blinded. High risk of bias indicates there was a clear indication of potential bias. Not applicable bias indicates that the source of bias was not relevant. For example, random allocation of animals when all animals received the same treatment at the same time.

Relatively few experiments adequately used and reported using techniques to reduce bias (**Fig 3**). Many experiments did not report randomizing animals to treatment groups (43%, n = 24). No experiments reported using techniques of sequence generation, allocation concealment, or blinded caregivers. Risk of bias was most commonly minimized by were blinding during outcome assessment (50%, n = 28), having complete outcome data or addressing missing outcome data (41%, n = 23), ensuring similar baseline characteristics between groups (16%, n = 9), and randomly assessing outcomes (14%, n = 8). The least used methods to reduce risk of bias were randomly housing rats throughout the room (9%, n = 5) and blinding investigators to treatments (7%, n = 4). Details of which outcome measures were blinded as well as specific reporting quality and risk of bias information for each experiment are included in **S2 Table**.

## Discussion

We conducted a systematic review to synthesize the empirical literature involving tickling rats. Our search procedure identified 32 articles with 56 experiments. An international and interdisciplinary field of researchers use tickling to accomplish a variety of objectives. Each article was reviewed to achieve three aims: (a) describe the characteristics of rat tickling, (b) summarize outcomes of rat tickling, and (c) evaluate factors moderating rat tickling outcomes. There are a variety of specific tickling methods used in various rat strains, ages, and housing. The most common tickling method was the original “Panksepp method”. Overall, tickling outcomes support positive welfare benefits of tickling for rats including increased production of positive vocalizations, improved approach behavior, decreased behavioral measures of anxiety and fear ins standardized tests, and improved handling. However, these outcomes of tickling can be modified by inter-individual differences, pharmacology, housing, genetics, age, timing, specific technique, and the presence of bedding. Overall tickling appears to be a promising welfare enhancing technique that also measures affect, but specific investigation of the best methods is needed.

### Characteristics of tickling

To achieve the first aim of our review, several elements of rat tickling were examined in each article, including terminology, rat sex, strain, and housing, tickling duration and frequency, and study design. Although fifteen different terms were used to describe the technique used, the word “tickling” was mentioned at least once in every article. Terminology is an important tool for communicating research to both others in academia and the public. Often, the original term for a procedure is used. However, the large variety of additional terms in this review seems to indicate a disagreement of the best term. Anecdotally, the term “tickling” has been critiqued based on the perception that it lacks scientific rigor by researchers outside the field, is unprofessional, and inadequately describes the procedure. Although the motion of the fingers used in tickling is similar to the motion used when tickling humans, it may or may not mimic human to human tickling. Rather, tickling rats mimics rat rough-and-tumble play by making contact with body areas that are stimulated during this type of play. Based on our review of the literature, we recommend using the term tickling at least once in each article, preferably in the abstract, for consistency across the field. If researchers wish to use an additional term we recommend either "heterospecific play” which was the second most common description and perhaps more accurately describes the interaction or “playful handling” which also describes the technique and is more easily understood by the general public. Consistent use of terminology may help advance knowledge more quickly and efficiently by allowing the technique to be more easily identified and recognized by both researchers and the public.

The tickling procedure varied across studies, although the most common method was to replicate the procedure developed by Panksepp and Burgdorf, with each tickling session lasting for 2 minutes total, with 1 minute of active tickling by alternating between 15 seconds of rest and 15 seconds of active tickling. Most frequently, tickling was conducted over 5 days. However, there was great diversity in session length, amount of time active tickling, and number of sessions. Reporting of other characteristics of the tickling procedure such as presence of bedding, light intensity, and the time of day tickling took place was poor. We recommend that future studies provide specific details about the tickling procedure since bedding increases positive vocalizations [17], and high light intensity decreases social play in juveniles [45]. We also recommend an experimental investigation of the ideal tickling duration and frequency. Until then, we recommend that researchers tickle for 2 minutes with 1 minute of active tickling for at least 5 sessions since this is the most common method.

Tickling procedures were applied to the four most common rat strains, both sexes, and across ages with typically short to no acclimation periods. Although the effect of rat strain on tickling outcome has not been evaluated, it is likely that rat strain impacts outcomes considering that different types of rats have different play behaviors [46]. There are also sex differences in rat play. Juvenile males engage in rough-and-tumble play fighting attacks more frequently than females [47]. Male rats also transition to a rougher form of playful defense post puberty [47]. Age is also an important factor, considering that rat play peaks between 30–40 days and declines as rats approach sexual maturity [48]. We recommend that, unless sufficient rationale can be given for an alternative, future research include both sexes, focus on juvenile rats, and if possible test multiple strains of rats with an adequate acclimation period.

### Outcomes of tickling

To achieve the second aim of our review–summarize outcomes of rat tickling–we reviewed studies that included a control for tickling. Overall, almost all studies that investigated tickling in rats reported positive welfare outcomes and that using tickling was beneficial in studying positive affective states. The most common finding was increased production of 50-kHz vocalizations, which are indicative of positive affect, and in turn improved welfare. Given that the tickling technique was specifically developed to study these vocalizations this result is unsurprising [16, 18]. The second most common finding of increased approach behavior is thought to relate to the positive reward value of the interaction which causes rats to want to move back to the hand for more tickling. Outcomes related to reduced generalized anxiety and fear of humans, as well as improved handling, also indicate the promising benefits of tickling to rat welfare. Finally, tickling was successfully used to study the genetic and cellular effects associated with positive affect.

Among the experiments that provided a control for tickling, five explicitly used tickling to induce or measure positive affective states. For example, since previously low hippocampal cell proliferation has been associated with depression, increasing hippocampal cell proliferation is essential for the therapeutic efficacy of anti-depressants [49]. However, it has been controversial whether hippocampal cell proliferation is a trait or state effect of this psychopathology. To investigate this trait vs state controversy, one experiment used tickling and individual differences in calling rate to show that hippocampal cell proliferation depends on an interaction between a pre-disposing trait and stimulation-depending variations in the subject’s affective state [19]. Additionally, a series of studies used tickling to model laughter in humans and study its effects on gene expression and neurogenesis in the brain and salivary glands [21, 29, 30]. Tickling is useful for studying positive affect because it can be used quickly, consistently and reliably in contrast to other tests such as cognitive bias or sucrose consumption. For example, counting 50-kHz vocalizations has been repeatedly shown to have higher inter- and intra- rater reliability and is easy to train.

Despite the described benefits from tickling, researchers should remain cautious when implementing this procedure widely across rat sexes, ages, and housing types. Single housing of rats less than 50 days old were used in 81% of experiments, and male rats were used in more than half of experiments. Therefore, some findings may be most relevant to singly housed juvenile male rats. Additionally, as several different control conditions were used and techniques to address bias were not sufficiently reported, different results may be seen with different comparison conditions. Finally, as shown below many variables have the potential to moderate tickling outcomes.

One area to consider in future research is the selection of appropriate control conditions. Eleven experiments used an “active” control that involved at least exposing control rats to a passive hand or light touch. The remaining experiments either gave control rats no additional manipulation or simply transferred them to the test box. Choice of control should be determined by experimenters explicitly depending on their experimental question. Stroking or light touch is aversive and therefore can serve as an aversive control [18]. Restraint on the back has been found to have mixed results compared to tickling and should be used with caution as a control. Transferring rats to the test box can be used to control for the novelty and reward of a new environment. However, if tickling is being investigated as a habituation method or method to elicit a positive affective state, minimal handling may be the best option.

A final area of concern related to rat tickling is that no experiments reported the impact of tickling rats on their human caretakers. Animal care personnel, particularly those involved in euthanasia, report mild to moderate traumatic general stress, higher level of work stress, and may have higher employee turnover [50]. To combat these effects, several universities have implemented programs to mitigate stress and compassion fatigue such as Washington State University’s Animal Caregiver Training. Tickling may help form and strengthen the human-animal bond by increasing direct care for the animals and helping caretakers recognize individual animals [51]. Establishing a human-animal bond, which is bolstered by human-animal interactions, can be beneficial for humans working with laboratory animals [51]. Therefore, it is important to consider caretaker outcomes, in addition to animal outcomes, when investigating human-animal interactions such as tickling.

### Tickling outcome moderation

To achieve the third aim of this review–evaluate factors moderating rat tickling outcomes–we reviewed experiments that specifically investigated factors that could moderate the outcomes of tickling. These experiments fell into five main categories: inter-individual differences in random bred rats, pharmacology, housing, genetics, and other. Overall our results indicate that care must be taken while applying tickling in the laboratory.

Inter-individual differences are one of the most relevant moderators of outcomes of tickling for facility personnel. Rats repeatedly show a large range of 50-kHz vocalizations in response to tickling that impact experimental outcomes. In one study, only high-calling rats showed a positive cognitive bias after tickling [8]; thus low-calling rats may experience tickling differently than high-calling rats. Further investigation is needed to determine if low-calling rats indeed find the tickling intervention positive, neutral, or negative. Since you can also bi-directionally select rats for either a high- or low-calling rate and find similar differences in line bred rats, it seems to indicate these are trait differences. In future studies, we strongly recommend that investigators include calling rate as a continuous covariate in all statistical models and determine the calling rate of control rats after the termination of the experiment. Tickling can be used to determine behavioral traits and subsequently how a trait response to tickling affects response to stress [52]. Selectively bred high-calling rats could also be useful for understanding the importance of playful joy [39].

Tickling rats to investigate the effects of pharmacological substances on positive affect has been used repeatedly. All experiments found differences in 50-kHz vocalizations after application of some substances. One article attempted to use tickling combined with the application of the psychotomimetic drug phencyclidine to model the negative symptoms of schizophrenia, but concluded that it was unclear if this model would be valid or not [53]. Another article successfully concluded that 50-kHz calls in response to tickling were mediated by dopamine release as evidenced by a decrease in calls after application of dopamine agonists [28]. This article was using tickling to model the importance of play behavior during adolescence. Finally, an additional article has used tickling to evaluate calling rates and drug administration to further investigate the effects of trait differences on chronic variable stress as a model of depressions [34]. Overall, tickling is a promising method to evaluate pharmacological compounds designed to improve psychological measures of affect while also considering the individual differences in calling rates.

One aspect of tickling moderation–housing–was investigated in the initial work by Panksepp and Burgdorf in 2000 and 2001. Their results showed that rats housed individually had more numerous positive vocalizations and reduced approach latencies. Socially housed animals actually learned to avoid tickling. However, the one study that investigated the impact of housing more recently did not find any significant differences, which was partially attributed to tickling group-housed animals with their cage mates in their home cage [23]. All experiments investigating the impact of housing differences were conducted in the same laboratory. An increasing amount of evidence indicates that environmental standardization within laboratories contributes to poor reproducibility of animal experimental outcomes [54]. Therefore, it would be beneficial for experimenters external to this laboratory to investigate housing factors, therefore increasing environmental heterogenization for reproducible results. Until then, we recommended researchers tickle group-housed rats in their home cage with cage mates.

There are a few additional factors that experimenters should keep in mind while tickling rats. Rats should be tickled before aversive procedures, rather than after, especially since stress decreases positive vocalizations during tickling. Bedding should be used in the tickling location whenever possible in order to increase reward value and number of 50-kHz vocalizations [41]. Bedding may increase the rats comfort while being turned over on their back. Additionally, it seems that tickling rats for the first time when they are adults may not be as rewarding as tickling rats as juveniles only or as both juveniles and adults. Investigators should tickle rats before stressful procedures, rather than after, since stress decreases 50-kHz vocalizations during tickling. Finally, considering the high rates of 22-kHz calls that are elicited while conducting the Schwarting tickling technique, we recommend that investigators rely on the standard Panksepp method of tickling.

### Reporting quality, risk of bias, limitations, and future directions

Poor reporting and use of methods to reduce risk of bias are a serious concern in the reviewed literature on tickling. Our reporting quality and risk of bias assessment demonstrate that crucial pieces of information are missing from many studies, such as randomization to treatment groups, random housing, and blinding during outcome assessment. Following guidance from the SYRCLE Risk of Bias tool, we coded studies as having an unclear risk of bias when insufficient details were reported to properly assess the risk of bias [14]. However, it is perhaps more likely that experiments did not report details because they simply did not mitigate these areas of potential bias. In this case, there is a risk that the effects of tickling and impact of tickling moderators have been overestimated and may be unable to be replicated [55]. Undoubtedly, more clear reporting is needed throughout experiments from sequence generation, random housing, and even clearly indicating that all animals were used in the analysis. Overall, we encourage authors to check their own articles using the SYRCLE tool before publishing, as well as follow the recommendations outlined by Macleod (2009) and the Animal Research: Reporting In Vivo Experiments (ARRIVE) Guidelines Checklist [56].

One limitation of this systematic review is that we did not include theses in our review. Therefore, our conclusions could be limited due to a publication bias or the file drawer problem, which occurs when negative or non-significant positive findings are placed in a file drawer rather than published. However, considering that some of the studies did include non-significant findings we consider this limitation to be a more minor concern. Overall, there is a need for future tickling studies to reduce risk of bias, report these efforts, and publish both significant and non-significant findings.

## Conclusion

Overall, this systematic review demonstrates many positive effects of tickling rats, including increased 50-kHz vocalizations, increased positive human approach behaviors, decreased fear and anxiety after stress, and decreased stress from handling. Some of these effects are stronger in high-calling tickled animals, indicating inter-individual differences in outcomes from tickling. A review of the characteristics of tickling indicate that a wide variety of techniques are used, with the most common being Panksepp’s original 2000 method. Future research is needed to determine the ideal dosage of tickling and the effect of calling rate on outcomes. Overall, we recommend tickling as a viable method for studying positive affective states in rats and improving rat welfare.

## Supporting information

S1 Checklist PRISMA checklist. (DOC)

S1 Text Full Search Strategy. (DOCX)

S1 Table Tickling Systematic Review Data Extraction by Experiment. (XLSX)

S2 Table Reporting Quality & Risk of Bias Assessment of Tickling Experiments. (XLSX)
