# Supplementary material for: Rat tickling: A systematic review of applications, outcomes, and moderators
Source: PLoS One. 2017 Apr 6;12(4):e0175320. doi: 10.1371/journal.pone.0175320 (PMC5383284; doi:10.1371/journal.pone.0175320)
Supplement: S1 Text — (DOCX) [file pone.0175320.s002.docx]

**S1 Text. Full Search Strategy**

("Playful handling" OR "Tickle" OR "Tickling" OR "Heterospecific play") AND ("Rat" OR "Rats")

This search was applied to:

1. Scopus
   - TITLE-ABS-KEY((playful handling) OR Tickle OR Tickling OR "Heterospecific play" OR "Hetero-specific play") AND TITLE-ABS-KEY(Rat OR rats)
2. PubMed
   - ("Playful handling" OR "Tickle" OR "Tickling" OR "Heterospecific play") AND ("Rat" OR "Rats")
3. Web of Science
   - TS=("Playful Handling" OR Tickling OR Tickle OR "Heterospecific play") AND TS=(Rat OR Rats)
   - Timespan=All years
   - Search language=Auto
4. PsychInfo
   - ("Playful Handling" OR Tickling OR Tickle OR "Heterospecific play") AND (Rat OR Rats) TI ( ("Playful Handling" OR Tickling OR Tickle OR "Heterospecific play") AND (Rat OR Rats) ) OR AB ( ("Playful Handling" OR Tickling OR Tickle OR "Heterospecific play") AND (Rat OR Rats) ) OR KW ( ("Playful Handling" OR Tickling OR Tickle OR "Heterospecific play") AND (Rat OR Rats) )
